# *Lactobacillus plantarum* L11 and *Lactobacillus reuteri* LR: Ameliorate Obesity via AMPK Pathway

**DOI:** 10.3390/nu17010004

**Published:** 2024-12-24

**Authors:** Shukun Liang, Jintao Sun, Xinshu Gu, Ya Zhao, Xiumin Wang, Hui Tao, Zhenlong Wang, Yougang Zhong, Jinquan Wang, Bing Han

**Affiliations:** 1 Key Laboratory of Feed Biotechnology, Ministry of Agriculture and Rural Affairs, Institute of Feed Research, Chinese Academy of Agricultural Sciences, Beijing 100081, Chinawangjinquan@caas.cn (J.W.); 2 School of Veterinary Medicine, China Agricultural University, Beijing 100193, China; zhongyougang@cau.edu.cn

**Keywords:** probiotics, lipid metabolism, gut microbiota, mechanism

## Abstract

Objectives: The purpose of this study was to find the potential mechanism of two Lactobacillus (*Lactobacillus plantarum* L11 and *Lactobacillus reuteri* LR) on ameliorating obesity, including lipid metabolism and gut microbiota. The two isolates have been studied to have good characterization in vitro, but in vivo studies in modulating lipid metabolism and gut microbiota were not studied. Methods: In this study, mice with HFD supplemented with L11 or LR exhibited slower obesity progression, including reduced weight gain, abdominal fat accumulation, liver damage, inflammation, and adipose lesions. Results: Total cholesterol (TC) and triglycerides (TG) in the serum were significantly reduced (*p* < 0.01). The inflammatory marker interleukin-6 (IL-6) notably decreased (*p* < 0.05). Both Lactobacillus strains altered the gut microbiota composition, increasing the relative abundance of *Alistipes* and *Lactobacillus*, while L11 also raised *Lachnospiraceae* abundance. Results of the Western blot analysis showed that L11 and LR influenced the PPAR and AMPK pathways. Conclusions: L11 and LR can effectively reduce obesity by modulating gut microbiota and activating the PPAR–AMPK pathway, leading to decreased liver injury and systemic inflammation in mice fed with an HFD. In the future, the two probiotics may provide a new way for clinically ameliorating obesity on human beings.

## 1. Introduction

Obesity, as a kind of nutritional metabolic disease, has widely existed in humans. There are just very limited drugs that can effectively treat this disease in humans, and other secondary diseases caused by obesity, such as diabetes [1], pancreatitis [2], and fatty liver, seriously damage health and life [3,4]. At present, controlled diets are often used to restrict body weight and reduce the occurrence rate of nutritional metabolic diseases, but the effect is not stable because of individual diversity [5].

Probiotics, as a kind of active bacteria, bring beneficial effects to the host [6]. Lactic acid bacteria are safe and most widely used as a kind of probiotics [7], and have a good effect on lipid modulation. They can reduce serum cholesterol content [8] and control lipid accumulation by adjusting lipid metabolism pathways [9,10]. *Lactobacillus plantarum* has an apparent effect on lipid reduction [11], but the mechanism was not mentioned. *Lactobacillus reuteri* was showed to reduce serum cholesterol, but the relationship with the pathway of AMPK was not mentioned [12]. These studies showed that Lactobacillus can modulate lipid metabolism.

However, the pathway of *Lactobacillus plantarum* and *Lactobacillus reuteri* on lipid metabolism in vivo has been less well elucidated using the HFD-fed mice model. Therefore, the purpose of this study was to provide an effective way for the clinical management of obesity and lipid disorders for the host. In this study, we tested the basic signs, blood lipids, liver functions, intestinal lipid content, and gut microbiota to find the effects and mechanism for ameliorating obesity.

## 2. Materials and Methods

### 2.1. Preparation of Lyophilized Probiotics for Animal Test

*Lactobacillus plantarum* L11 and *Lactobacillus reuteri* LR used in this experiment were preserved at −80 °C in our lab and deposited in the General Microbiology Center of China Council for the Preservation (Chaoyang District, Beijing, and the Institute of Microbiology, China Academy of Sciences). The deposit number of the *Lactobacillus plantarum* L11 is CGMCC24558, and that of the *Lactobacillus reuteri* LR is CGMCC NO.27590. Both probiotics were cultured using De Man–Rogosa–Sharpe (MRS, BD Difco, Sparks, MD, USA) agar at 37 °C for 24 h. The cultures were centrifuged at 6000 rpm at 4 °C for 20 min. Afterward, a mixture of 20% skimmed milk powder, 1% sucrose, and 1% galactose was used as the protective agent for the preparation of lyophilized probiotics. The viable count of lyophilized probiotics was 10^12^CFU/g and was stored at −80 °C for testing.

### 2.2. Animals and the Experiment Design

All of animal experiments were approved by the Laboratory Animal Ethical Committee and its Inspection of the Institute of Feed Research of China Academy of Agricultural Sciences (IFR-CAAS-20230430). Twenty-four male (six-week-old, healthy) BAL b/c mice were raised at 22 ± 1 °C, 55 ± 10% humidity, and 12 h of light/dark cycle on the base of CAAS. After one week of adaptation, the mice were randomly divided into four groups (n = 6/group). The treatment groups were L11 (HFD with *Lactobacillus plantarum* L11, 10^9^ CFU/mouse), LR (HFD with *Lactobacillus reuteri* LR, 10^9^ CFU/mouse), M (HFD model without probiotics), and CK (Control, normal diet without probiotics). The experiment was carried out for 60 days. All animals received free access to water and food. Antibiotics were not used all through the test. Groups L11, LR, and M were fed with HFD (60% fat, 20% protein, 20% carbohydrate), and the CK group was fed with normal diet (5% fat, 18% protein, 77% carbohydrate). Groups L11 and LR received the probiotics each day, and groups M and CON only received the sterile PBS each day. The hair was observed each day. After continuous administration for 60 days, the mice were fasting for 12 h and euthanized after blood collection from the eyes. A part of liver and colon samples was rapidly frozen in liquid nitrogen and then transferred to −80 °C for cryopreservation. In addition, stool samples, blood samples, a portion of abdominal fat samples, duodenum samples, and the remaining bowel segments were all frozen at −20 °C. The other part of liver, fat, and intestinal samples was immersed in tissue fixative and stored at 4 °C.

### 2.3. Blood Biochemical Test

Blood samples were collected using EDTA tubes without anticoagulants, and biochemical parameters were measured using a biochemical analyzer (MNCHIP Technology Co., Ltd., Tianjin, China). The analysis included total protein (TP), blood urea nitrogen (BUN), gamma-glutamyl transferase (GGT), albumin (ALB), globulin (GLO), creatine kinase (CK), and aspartate aminotransferase (AST), among others.

### 2.4. ELISA

The serum and tissue samples were tested. The jejunum, liver, and feces samples were extracted in a RIPA lysis buffer and centrifuged at 8000 rpm for 2 min. The homogenate samples were centrifuged at 12,000 rpm for 3 min at 4 °C, and the supernatant was stored at 4 °C. Inflammatory cytokines (TNF-α and IL-6), sIgA, and lipid metabolism-related indicators (TC, TG, HDL-C, LDL-C, and NPC1L1) were detected by an ELISA kit (Jiangsu Meimian Industrial Co., Ltd., Yancheng, China) with an OD450 nm value. Then, the samples TNF-α, IL-6, sIgA, TC, TG, HDL-C, LDL-C, and NPC1L1 were all determined with an OD450 nm value.

### 2.5. Organizational Analysis

The liver tissue, jejunum, and abdominal fat samples were fixed in tissue fixative, and the sections were sectioned at 4 μm and roasted at 65 °C for 15 min. Xylene A solution was put in sequence for 1 min, and xylene B solution was cut into slices for dewaxing after 10 min. Then, 100% ethanol, 95% ethanol for 1 min, 80% ethanol for 1 min, and 70% ethanol for 1 min were implemented in sequence, and xylene was eluted with water. Hematoxylin dye solution was added to the samples for 3 min and washed with water. One percent hydrochloric acid alcohol was added for 30 s and washed with water. One percent ammonia was added for 30 s and washed with water. The method used was referred to the reference [13].

### 2.6. Quantitative Real-Time PCR

Total RNA was extracted from liver using Trizol (Ambion, Austin, TX, USA) and subsequently reverse-transcribed with a Maxima H Minus First Strand cDNA Synthesis Kit (Thermo Scientific, Waltham, MA, USA). Quantitative real-time PCR was conducted using the EcoTM Real-Time PCR System (Illumina, San Diego, CA, USA). The reactions contained 20 ng of cDNA, KAPA SYBR^®^ FAST qPCR Master Mix (Kapa Biosystems, Wilmington, MA, USA), and specific primers for the target genes, with annealing temperatures as listed in Table 1. Gene expression levels were normalized to β-actin using the ΔΔCt method and are presented as mean ± SEM.

### 2.7. Western Blot Analysis

Liver samples were lysed in a RIPA buffer supplemented with a protease/phosphatase inhibitor cocktail, followed by centrifugation. Protein concentrations in the supernatants were determined by a BCA protein assay kit (BOSTER, Wuhan, China). Equal amounts of protein (100 μg) from the tissue samples were resolved via sodium dodecyl sulfate-polyacrylamide gel electrophoresis (SDS-PAGE) and subsequently transferred onto a nitrocellulose membrane (GE Healthcare Life Sciences, Marlborough, MA, USA). The membrane was then blocked with 5% skim milk for 1 h and incubated overnight at 4 °C with rabbit anti-mouse primary antibodies targeting p-AMPK (CST, Danvers, MA, USA), AMPK (CST, USA), and β-actin (1:1000) (CST, USA). Washed with Tris-buffered saline (TBST, 0.05% Tween 20 contained), the membranes were incubated with HRP-conjugated goat anti-rabbit secondary antibodies (1:5000) for 1 h at room temperature. Protein bands were visualized using Western Lightning™ Chemiluminescence Reagent Plus (PerkinElmer Spain SL, Madrid, Spain) and detected with the Tanon imaging system. Band intensity was quantified through densitometric analysis using ImageJ software 1.53K (Free Software Foundation Inc., Boston, MA, USA).

### 2.8. PCR Amplication for Gut Microbiota

Microbial genomic DNA was extracted from 20 colon content samples using an E.Z.N.A. Mag-Bind Soil DNA Kit (Omega, M5635-02, Norcross, GA, USA), and DNA concentration was measured with a Quibit dsDNA HS Assay Kit (Thermo, Waltham, MA, USA). The forward primer used for PCR was CCTACGGGNGGCWGCAG, and the reverse primer was GACTACHVGGGTATCTAATCC. The PCR conditions was consulted to the reference [13]. The extracted DNA was sequenced for 16S rDNA at Sangon Biotech (Shanghai) Co., Ltd. (Shanghai, China).

### 2.9. Data Analysis

Gut microbiota analysis was conducted using QIIME 2 and R version 3.5.1. Alpha diversity indices and bacterial abundance were compared across groups using the Kruskal–Wallis test, followed by pairwise Mann–Whitney U tests with Bonferroni correction. Alpha diversity was evaluated using the Shannon index, while β-diversity was assessed through UniFrac distance metrics and visualized via principal coordinate analysis (PCoA). Differences in bacterial abundance were identified with STAMP (version 2.1.3) and LefSe (version 1.1.0). Correlation coefficients and *p*-values for communities/OTUs were calculated using SparCC (version 1.1.0). Results, excluding 16S rRNA gene data, are presented as mean ± SEM. Statistical analyses were performed with IBM SPSS 19.0 using Student’s *t*-test for two-group comparisons and one-way ANOVA with Newman–Keuls post hoc tests for multiple groups. A *p*-value of less than 0.05 was considered significant (* *p* < 0.05, ** *p* < 0.01, *** *p* < 0.001).

## 3. Results

### 3.1. Lactobacillus Reduced Fat Accumulation in HFD Mice

**Figure 1:**
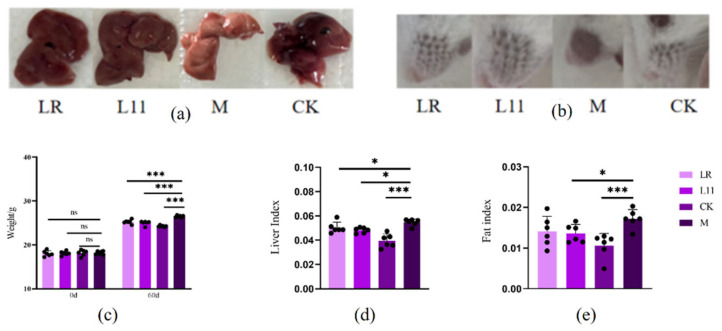
Changes in basic signs in HFD mice. (**a**) Liver condition of mice on 60th day. (**b**) Condition of hair loss in mice on the 60th day. (**c**) Mice weight on the 0th and 60th day. (**d**) Liver index of mice on the 60th day. (**e**) Fat index of mice on the 60th day. * *p* < 0.05. *** *p* < 0.001. ns meant no siginificance.

After 16 weeks of HFD feeding, basic signs and relevant blood biochemical indices were examined. Body weights of Group M were higher than groups CK, LR, and L11 (*p* < 0.001) (Figure 1c), and obesity index and liver index were much higher in group M compared with CK (*p* < 0.001) (Figure 1a–e). It is worth noting that the obesity index and the liver index were significantly different between LR and M (*p* < 0.05), and the liver index of mice fed with L11 significantly decreased compared to that of group M (*p* < 0.05). Furthermore, considering that obesity might affect the liver and kidney function of the body [14,15], we tested the blood biochemical indicators and found that the GGT of L11 was higher than that of M (*p* < 0.05). And the AST of LR was significantly decreased (*p* < 0.05). Furthermore, the GLO content of HFD mice fed with Lactobacillus was lower than that of M (*p* > 0.05), and the A/G value was increased (*p* > 0.05). As for kidney-related indicators, the BUN content of M was increased, and L11 can slow down this process (*p* < 0.05); the Mg content of M was also significantly increased, and significant differences were shown between LR and M (*p* < 0.05). In addition, the administration of LR and L11 also reduced the content of AMY and GLO (*p* < 0.05) (Table 2).

Therefore, LR and L11 can decrease body weight growth, abdominal fat accumulation, liver function, and kidney function of HFD mice and affected the carbohydrate metabolism.

### 3.2. Lactobacillus Reduced Lipid Content in HFD Mice

**Figure 2:**
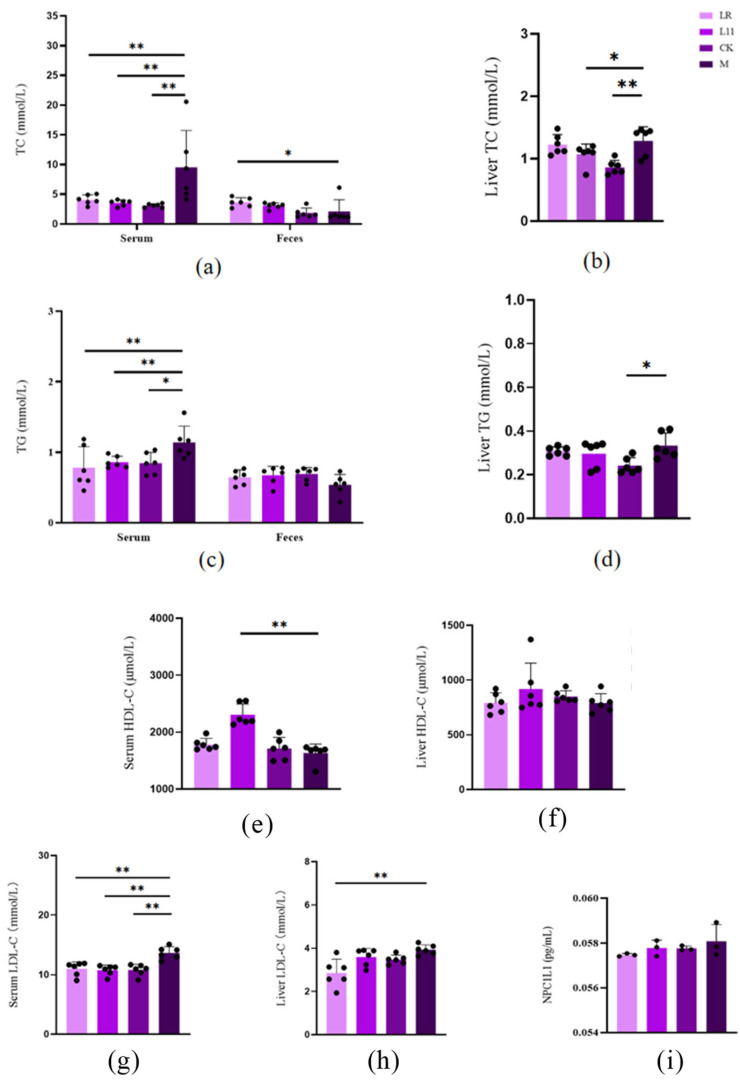
Changes in lipid content in HFD mice. (**a**) Serum and feces TC on the 60th day. (**b**) Liver TC on the 60th day. (**c**) Serum and feces TG on the 60th day. (**d**) Liver TG on the 60th day. (**e**) Serum HDL-C on the 60th day. (**f**) Liver HDL-C on the 60th day. (**g**) Serum LDL-C on the 60th day. (**h**) Liver LDL-C on the 60th day. (**i**) Intestinal HDL-C on the 60th day. * *p* < 0.05. ** *p* < 0.01.

It has been proved that mice fed with HFD showed significantly higher serum lipid levels, including TC, TG, and LDL-C [16]. In this study, we found that after the supplementation of Lactobacillus, the mice receiving the supplementation of Lactobacillus showed significantly lower serum cholesterol, triglyceride, and LDL-C contents than M (*p* < 0.01) (Figure 2a,c,e,g); it also showed lower liver cholesterol, triglyceride, and LDL-C contents, and the liver LDL-C of LR was significantly decreased (*p* < 0.01) (Figure 2b,d,f,h); the liver TC of L11 was significantly decreased (*p* < 0.05). With regard to feces, LR and L11 both showed higher TC and TG (*p* > 0.05) (Figure 2a,c). With regard to the intestine, we found that the content of NPC1L1 in the jejunum of M was increased (*p* > 0.05) (Figure 2i), but after supplying Lactobacillus, the expression of NPC1L1 decreased but was not significant (*p* > 0.05).

Therefore, LR and L11 can reduce the lipid content of HFD mice and accelerate lipid metabolism.

### 3.3. Lactobacillus Reduced the Inflammation in HFD Mice

**Figure 3:**
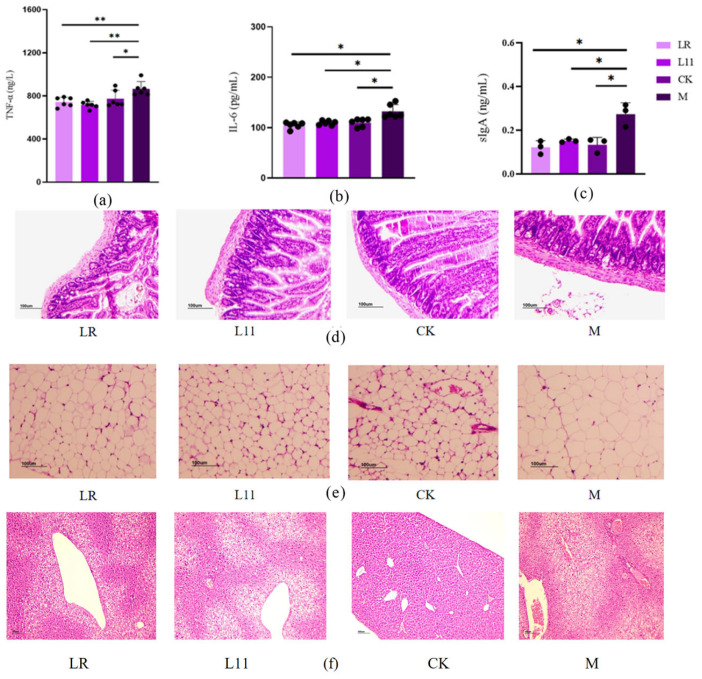
Inflammatory factors and histopathological changes. (**a**) Serum TNF-α on the 60th day. (**b**) IL-6 on the 60th day. (**c**) Intestinal sIgA on the 60th day. (**d**) Histopathological section of jejunum on the 60th day. (**e**) Histopathological section of fat issue on the 60th day. (**f**) Histopathological section of liver tissue on the 60th day. * *p* < 0.05. ** *p* < 0.01.

Obesity causes increased inflammation levels in the body [17]. We detected the inflammation-related factors in serum and found that the levels of inflammatory factors in M were significantly increased, including serum TNF-α, IL-6, and intestinal sIgA (*p* < 0.05) (Figure 3a–c). After the supplementation of LR and L11, the levels of inflammatory factors in mice were significantly reduced (*p* < 0.05). TNF-α was extremely significantly reduced (*p* < 0.01); IL-6 and intestinal sIgA were significantly reduced compared with those in M (*p* < 0.05).

The jejunum tissue showed that the intestinal mucosal structure in M was defective, the intestinal glands were significantly swollen and thick, and the intestinal crypts were enlarged. In addition, the intestinal villi were fractured, the length was significantly shorter, and the serosal layer was significantly thickened.

In adipose tissue, fat cells undergo massive hypertrophy and hyperplasia, and some fat cells were distributed in a mass manner, with large differences in size, and inflammatory cell infiltration and fat cell necrosis can be seen. LR and L11 can alleviate this phenomenon, and it can be clearly observed that the intestinal serosal layer was relatively thin in thickness, and the gland swelling was alleviated. The structural integrity of intestinal mucosa and the integrity of intestinal villi were improved. In adipose tissue, lipid droplet deposition was obviously weakened, with uniform adipocyte size and significantly reduced inflammatory cells (Figure 3d–f). The number of adipocytes in the M group was significantly reduced, and there were notable abnormalities in the arrangement of hepatic cords, with blood vessels generally lacking a circular shape. In Figure 3e, plenty of fat cells became hypertrophic, even with more inflammatory cells compared with other groups. In Figure 3f, liver necrocytosis was apparently observed in the M group, while L11 and LR could reduce the necrocytosis degree.

Therefore, LR and L11 can reduce the inflammatory response in vivo of mice fed with an HFD, including inflammation of intestinal and adipose tissue.

### 3.4. Lactobacillus Reduced the Expression of Liver Lipid-Related Proteins in HFD Mice

**Figure 4:**
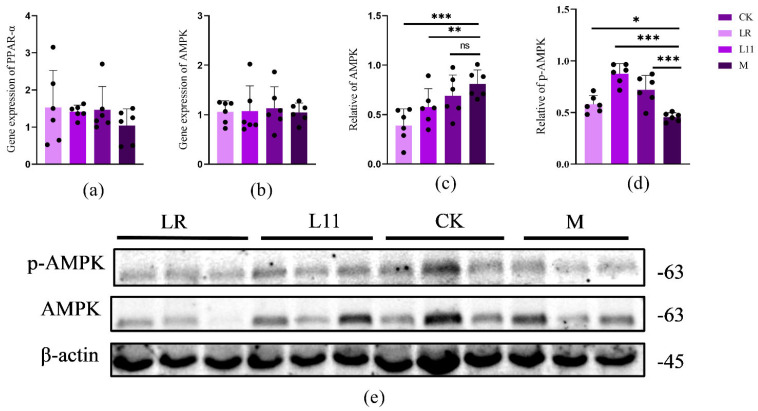
Inflammatory factors and histopathological changes. (**a**) The expression of liver PPARα in mice on the 60th day. (**b**) The expression of liver AMPK in mice on the 60th day. (**c**) Quantification of Western blot analysis showed that LR and L11 changed the expression levels of AMPK (n = 6). (**d**) Quantification of Western blot analysis showed that LR and L11 changed the expression levels of p-AMPK (n = 6). (**e**) The protein expression levels of AMPK, p−AMPK, and β−actin (n = 6). * *p* < 0.05. ** *p* < 0.01. *** *p* < 0.001. ns meant no significance.

In order to identify the specific pathway of LR and L11 affecting lipid metabolism in mice, the Western blot method was used to determine proteins related to lipid metabolism in mice’s liver. It was found that compared with M group, LR and L11 increased the phosphorylation expression of p-AMPK and decreased the expression of AMPK in mice liver (*p* < 0.01) (Figure 4c–e). At the same time, the qPCR method was used to determine the proteins related to lipid metabolism in the liver of mice. The expression of AMPK in the liver of HFD mice fed with Lactobacillus was decreased, and the content of PPAR−α had no significant difference (*p* > 0.05) (Figure 4a,b).

Therefore, LR and L11 can increase the lipid metabolism of HFD mice, mainly affecting the lipid metabolism mediated by the PPAR, which mainly acts on the PPAR-α in the PPAR pathway and is indirectly affected by the AMPK pathway.

### 3.5. Lactobacillus Improved Intestinal Microbiota in HFD Mice

**Figure 5:**
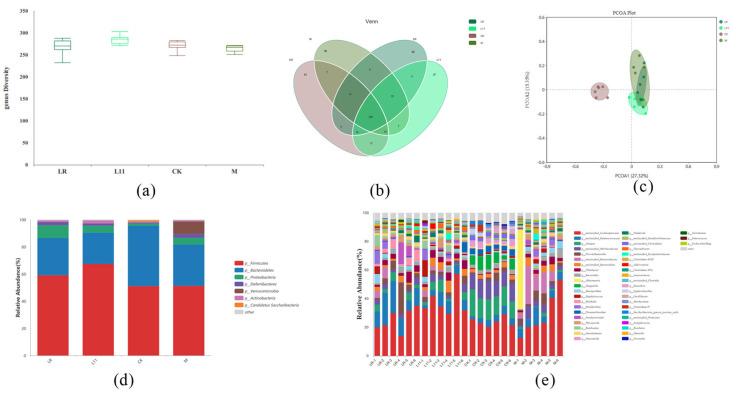
Changes in intestinal microbiota in mice. (**a**) α−Diversity of colonic content of mice on the 60th day. (**b**) Venn diagram showed the number of microbiota in mice colonic content on the 60th day. (**c**) PCoA diagram showed the β−diversity in mice colonic content on the 60th day. (**d**) Relative abundance of microbiota in mice colonic content on the 60th day (phylum level). (**e**) Relative abundance of microbiota in mice colonic content on the 60th day (genus level).

The α-diversity of intestinal microbiota was analyzed, and it was found that feeding LR and L11 can enhance the α-diversity of HFD mice intestinal microbiota (*p* > 0.05) (Figure 5a). From the Venn diagram, 268 genera of bacteria were common to all groups, and there was no significant difference in the number of microbiota in mice (*p* > 0.05) (Figure 5b). The β−diversity of intestinal microbiota revealed the differences in microbiota in each group. The PCoA diagram showed that the intestinal microbiota in HFD mice was significantly different from other groups (Figure 5c). However, LR and L11 changed the intestinal microbial composition in mice fed with HFD and made mice show a higher similarity with CK.

According to previous studies, mice microbiota mainly consists of Firmicutes and Bacteroides [18,19]. Research showed that HFD may increase the abundance of Verrucomicrobia and decrease the abundance of Bacteroidetes, and also increase the ratio of Firmicutes/Bacteroidetes, which was similar to previous studies [20]. To identify changes in specific microbiota, the phylum diversity of microbiota in each group was compared, and it was found that exogenous administration of Lactobacillus changed the relative abundance of specific microbiota in HFD mice (Figure 5d). On the phylum level, the abundance of Verrucomicrobia was significantly reduced in LR and L11 compared with M (*p* < 0.05). On the genus level, the microbiota of LR and L11 was changed, and among the first 10 changed genera, Alistipes and Prevotella were relatively more abundant (Figure 5e). And the number of Bacteroidetes in M was significantly reduced compared with CK (*p* < 0.05). LR mainly affected the abundance of Proteobacteia in the intestinal tract (*p* < 0.05), and it can significantly increase the content of Proteobacteia and also increase the number of Phocaeicola. L11 mainly affected the abundance of Firmicutes in the intestinal tract fed with HFD (*p* < 0.05).

## 4. Discussion

In this study, we found that LR and L11 improved the health status and reduced the incidence of obesity in HFD mice. This effect may occur through the action of LR and L11 on the in vivo pathways of lipid metabolism and glycogenolysis [21,22,23,24]. In the present study, a series of effects of two lactic acid bacteria on obesity were investigated, including the impact on the liver, adipose, and small intestine microbiota of mice.. We also explored the correlation between HFD and liver lipid composition and the connection between gut microbiota and liver lipid composition.

HFD is characterized by a high-fat content, which leads to an abnormal accumulation of lipids in the body and ultimately to the development of obesity [25]. The main manifestations are weight gain, fat accumulation, and changes in the fat content of the blood and tissues of the organism [26], which ultimately leads to the development of other diseases [27]. In this study, LR and L11 were found to slow body weight gain and cause a significant reduction in abdominal fat and hepatic lipid deposition in HFD mice. In addition, biochemical assays of serum showed that LR and L11 were beneficial for the liver and kidney health of HFD mice; they can reduce albumin levels, and lower blood albumin and globulin levels may be indicative of hepatic hypoplasia [28,29]. HFD affects liver and body health through its effects on glucose and lipid metabolism [30]. We found that the mice fed with LR and L11 had lower glucose, amylase, AST and BUN levels in the serum. This indicated that LR and L11 can reduce the harm of HFD affecting liver function, kidney function, and enzymes related to glucose metabolism, and reduce the rate of kidney failure, diabetes, pancreatitis, similar to those mentioned in previous studies [31,32,33,34,35].

Further analysis of lipid metabolism in blood and feces showed that HFD increased the serum levels of TC, TG, and LDL-C and decreased the fecal levels of TC, TG, and LDL-C; these results are similar to those in previous studies [36,37]. Furthermore, the results indicated that LR and L11 can accelerate the lipid metabolism of mice under HFD and help to excrete cholesterol and triglycerides from the body instead of accumulating in the body. NPC1L1 was a key protein for cholesterol absorption and uptake, and it played a key role in the lipid metabolism of the body; the level of NPC1L1 was closely related to lipid transport, and the decrease in NPC1L1 would block the lipid metabolism [38,39]. So, we explored the effect of LR and L11 on NPC1L1, and the results showed that LR and L11 can inhibit the expression of the lipid absorption-related protein NPC1L1 in the gut by inhibiting the intracellular transport of cholesterol and reducing the absorption of cholesterol from food and bile [40].

PPAR is a transcription factor involved in several metabolic pathways [41]. PPAR regulates the expression of many target genes involved in intra- and extracellular lipid metabolism and is also involved in lipid cell differentiation, which leads to weight loss and increased insulin sensitivity in animals. Meanwhile, it has been shown that obesity leads to adipocyte dysfunction, and PPAR-α agonists can effectively restore adipocyte function in obese animals, thus controlling obesity occurrence [42]. And PPAR-α also has an effect on the inflammatory response of the gut microbiota, and the lack of PPAR-α leads to dysregulation of the gut microbiota, increased expression of inflammatory cytokines, and increased susceptibility to inflammation in mice [43]. In this study, we found that LR and L11 can modulate the PPAR pathway and inflammation levels by increasing the levels of PPAR-α in the liver. AMPK is a metabolism-related substance in animal organisms that plays a key role in regulating anabolic and catabolic metabolism, and it is also a core pathway in diabetes and obesity-related diseases. Activation of the AMPK pathway can ameliorate the metabolic imbalance induced by obesity and other diseases, and help the body keep healthy [44,45]. We found that LR and L11 can mediate lipid metabolism by affecting the AMPK pathway, and can increase the phosphorylation of AMPK protein expression in the body of HFD mice. Above all, LR and L11 can regulate HFD mice’s energy metabolism by affecting the PPAR–AMPK pathway.

HFD may trigger the increased levels of inflammation in the body, which may lead to other diseases [46,47]. Therefore, mice were tested for inflammatory factors in the blood, adiposity, and intestinal inflammation. It was found that HFD significantly induced systemic inflammation in mice, including leading to significantly elevated levels of IL-6 and TNF-α in the blood, as well as significantly elevated intestinal sIgA and destruction of intestinal tissue and gland enlargement, inflammatory cell infiltration of adipose tissue, and adipocyte necrosis [48,49,50]. In contrast, LR and L11 significantly ameliorated this phenomenon, with a significant reduction in serum inflammatory factors and tissue inflammation. All these phenomena suggested that these two Lactobacillus are effective in inhibiting HFD-induced inflammation, but the specific anti-inflammatory pathways require further investigation.

LR and L11 also affected the gut microbiology of HFD mice; the HFD-induced reduction in α-diversity and differences in gut microbiota were reversed by LR and L11, and feeding Lactobacillus increased the proportion of Firmicutes and Bacteroides of gut microbiota, which helped maintain the stability of the gut microbiota. In addition, feeding Lactobacillus reduced the number of Verrucomicrobia in HFD mice, which is associated with gut lipid absorption [51]. The relative abundance of Alistipes and Prevotellamassilia was higher, and both bacterial genera had a positive effect on lipid metabolism, with L11 also causing an increase in Lachnospiraceae intestinalis, a bacterium important for carbohydrate metabolism and fiber digestion [52]. In summary, LR and L11 mainly affect the PPAR–AMPK pathway and influence gut lipid absorption by affecting the gut microbiota, further regulating lipid metabolism and lowering body weight. We observed that HFD can affect the lipid content of mice, but what kind of effect remains unclear.

In this study, some of the changes in the bacterial microbiota were found to be different from those induced by feeding Lactobacillus to mice. However, these changes still slowed down the obesity process induced by HFD probably by PPAR–AMPK pathway. Also, only the mechanism in mice was studied in the research, since gut microbiota in mice is different from that in human beings; more research can be clinically implemented for its applicability in the future.

## 5. Conclusions

*Lactobacillus reuteri* LR and *Lactobacillus plantarum* L11 assisted mice to resist HFD, improved the condition of the mice liver, regulated lipid absorption, attenuated inflammation, maintained intestinal microbiota stability, regulated hepatic lipid metabolism, and corrected HFD-induced metabolic disorders; it can be concluded that *Lactobacillus* may generate a positive effect via the AMPK pathway in HFD model. Further research for more application objects of probiotics is needed in future.

## Figures and Tables

**Table 1:** Primer sequences of the target genes and annealing temperature.

Gene	Organism	Sequence5’~3’	AnnealingT °C
PPARalpha	Liver	FW:CAAGTGCCTGTCTGTCGGG RV: GCGGGTTGTTGCTGGTCT	50
AMPK	Liver	FW:CGTCGCCTACCACCTCATC RV: ATTTTGCCTTCCGTACACCTT	50
Beta-actin	Liver	FW:GTGCTATGTTGCTCTAGACTTCG RV: ATGCCACAGGATTCCATACC	50

**Table 2:** Blood biochemistry of HFD mice.

Biochemical Indicator	Group *			
	LR	L11	CK	M
Mg	1.10 ± 0.09 ^b^	1.25 ± 0.05 ^ab^	1.20 ± 0.06 ^ab^	1.32 ± 0.23 ^a^
Ca × P	57.83 ± 8.04	60.83 ± 7.65	64.83 ± 8.04	54.83 ± 30.76
P	2.19 ± 0.17	2.37 ± 0.29	2.52 ± 0.16	2.35 ± 0.69
Ca	2.12 ± 0.16	2.07 ± 0.04	2.08 ± 0.14	2.10 ± 0.09
tCO2	16.50 ± 1.87	15.83 ± 0.75	16.67 ± 1.21	16.00 ± 1.10
BUN/CRE	43.83 ± 12.44	30.50 ± 10.56	37.17 ± 18.53	44.83 ± 24.23
BUN	6.94 ± 0.79 ^ab^	5.68 ± 0.26 ^c^	6.06 ± 0.66 ^bc^	7.73 ± 1.04 ^a^
CRE	43.00 ± 16.35	51.00 ± 17.56	48.33 ± 21.57	52.83 ± 24.21
GLU	5.72 ± 0.87 ^b^	4.28 ± 0.57 ^c^	7.32 ± 1.20 ^a^	4.92 ± 0.81 ^bc^
AMY	936.67 ± 106.15 ^c^	1218.83 ± 68.71 ^ab^	1129.67 ± 106.67 ^b^	1342.33 ± 127.80 ^a^
CK	124.83 ± 67.80 ^b^	180.17 ± 89.95 ^ab^	342.83 ± 236.18 ^a^	135.83 ± 63.13 ^b^
TBA	3.18 ± 1.32	3.50 ± 1.26	3.87 ± 2.63	1.98 ± 0.35
ALP	86.67 ± 12.08	109.67 ± 20.77	116.33 ± 31.44	92.33 ± 12.50
GGT	1.25 ± 0.58 ^ab^	1.5 ± 0.34 ^a^	0.83 ± 0.54 ^ab^	0.70 ± 0.51 ^b^
AST/ALT	4.44 ± 1.07	5.98 ± 1.51	3.76 ± 0.64	5.21 ± 2.77
AST	104.83 ± 9.93 ^b^	154.83 ± 36.81 ^a^	126.67 ± 42.25 ^ab^	129.33 ± 35.41 ^ab^
ALT	24.67 ± 5.82	27.00 ± 9.30	33.00 ± 5.73	31.00 ± 16.60
TBIL	3.11 ± 0.60	3.68 ± 1.01	3.33 ± 1.11	2.71 ± 1.21
A/G	1.43 ± 0.05 ^b^	1.45 ± 0.054 ^b^	1.58 ± 0.075 ^a^	1.40 ± 0.06 ^b^
GLO	22.42 ± 3.58 ^ab^	22.6 ± 1.056 ^ab^	21.03 ± 1.30 ^b^	23.10 ± 0.87 ^a^
ALB	32.38 ± 2.11	32.60 ± 0.72	33.48 ± 1.59	32.73 ± 1.94
TP	54.80 ± 3.58	55.20 ± 1.06	54.52 ± 2.71	55.83 ± 2.72

* Mean ± SEM. ^abc^ The same letter on the same row of data means that the difference is not significant, and different letters mean that the difference is significant (*p* < 0.05). Abbreviations: Mg, magnesium; P: phosphorus; Ca: calcium; BUN, blood urea nitrogen; CRE, creatinine; GLU, glucose; AMY, amylase; CK, creatine kinase; TBA, total bile acid; ALP, alkaline phosphatase; GGT, γ-glutamyl transpeptidase; AST, aspartate aminotransferase; ALT, alanine aminotransferase; TBIL, total bilirubin; GLO, globulin; ALB, albumin; TP, total protein.

## Data Availability

The data are contained within the article.
